# Dissemination of *Metarhizium anisopliae *of low and high virulence by mating behavior in *Aedes aegypti*

**DOI:** 10.1186/1756-3305-4-171

**Published:** 2011-09-09

**Authors:** Filiberto Reyes-Villanueva, Javier A Garza-Hernandez, Alberto M Garcia-Munguia, Patricia Tamez-Guerra, Annabel FV Howard, Mario A Rodriguez-Perez

**Affiliations:** 1 Laboratorio de Biomedicina Molecular, Centro de Biotecnología Genómica, Instituto Politécnico Nacional, Boulevard del Maestro S/N esquina Elías Piña. Col. Narciso Mendoza, 88710, Cd. Reynosa, Tamaulipas, México; 2 Departamento de Parasitología Agrícola, Universidad Autónoma Chapingo, Texcoco, 56230, Estado de México, México; 3 Unidad de Formulados, Laboratorio de Inmunología, Facultad de Ciencias Biológicas, Universidad Autónoma de Nuevo León. Pedro de Alba S/N Ciudad Universitaria, 66450, San Nicolás de los Garza, Nuevo León, México

**Keywords:** autodissemination, sexual transmission, mating behavior, vector, virulence, Aedes aegypti, Metarhizium anisopliae

## Abstract

**Background:**

Dengue is a viral disease transmitted by *Aedes *mosquitoes. It is a threat for public health worldwide and its primary vector *Aedes aegypti *is becoming resistant to chemical insecticides. These factors have encouraged studies to evaluate entomopathogenic fungi against the vector. Here we evaluated mortality, infection, insemination and fecundity rates in *A. aegypti *females after infection by autodissemination with two Mexican strains of *Metarhizium anisopliae*.

**Methods:**

Two *M. anisopliae *strains were tested: The Ma-CBG-1 least virulent (lv), and the Ma-CBG-2 highly virulent (hv) strain. The lv was tested as non mosquito-passed (NMP), and mosquito-passed (MP), while the hv was examined only as MP version, therefore including the control four treatments were used. In the first bioassay virulence of fungal strains towards female mosquitoes was determined by indirect exposure for 48 hours to conidia-impregnated paper. In the second bioassay autodissemination of fungal conidia from fungus-contaminated males to females was evaluated. Daily mortality allowed computation of survival curves and calculation of the LT_50 _by the Kaplan-Meier model. All combinations of fungal sporulation and mating insemination across the four treatments were analyzed by χ^2^. The mean fecundity was analyzed by ANOVA and means contrasted with the Ryan test.

**Results:**

Indirect exposure to conidia allowed a faster rate of mortality, but exposure to a fungal-contaminated male was also an effective method of infecting female mosquitoes. All females confined with the hv strain-contaminated male died in fifteen days with a LT_50 _of 7.57 (± 0.45) where the control was 24.82 (± 0.92). For the lv strain, it was possible to increase fungal virulence by passing the strain through mosquitoes. 85% of females exposed to hv-contaminated males became infected and of them just 10% were inseminated; control insemination was 46%. The hv strain reduced fecundity by up to 99%, and the lv strain caused a 40% reduction in fecundity.

**Conclusions:**

The hv isolate infringed a high mortality, allowed a low rate of insemination, and reduced fecundity to nearly zero in females confined with a fungus-contaminated male. This pathogenic impact exerted through sexual transmission makes the hv strain of *M. anisopliae *worthy of further research.

## Background

*Aedes aegypti *is the primary vector of at least three relevant viral diseases [1] and has a cosmopolitan distribution. It is highly competent for dengue and its ability to breed in small contained water bodies makes it challenging to control [2]. In addition, insecticide resistance is reducing the ability of insecticides to control mosquito vectors. Therefore new tools that can tackle insecticide-resistant disease vectors are required. Several studies have evaluated the entomopathogenic fungus *Metarhizium anisopliae *against larvae of various mosquito species [3,4] and the adults of *Anopheles gambiae s.s*. and *Culex quinquefasciatus *[5]. Subsequent studies have confirmed that this pathogen decreases the competence of *An. gambiae *in malaria transmission [6] and causes high mortality rates in the adult dengue vectors, *A. aegypti *and *Aedes albopictus *[7-9]. However, in these studies the mosquitoes were infected by exposure to surfaces treated with oil-formulated or dry conidia. We are interested in exploring the polygamic behavior of male *A. aegypti *and the potential to spread conidia among females. A virgin male is capable of copulating with up to thirty females during the first thirty minutes of confinement [10] but they inseminate only the first five to seven ones [11]. In our previous study [12] we confined twenty *A. aegypti *females with lone virgin males that were contaminated with conidia of two highly virulent strains of *Beauveria bassiana*. After fifteen days 90% of females were infected, of which 50% died within seven days, and for survivors that laid eggs, fecundity was reduced by 96% in comparison with the control. Continuing with this line and to confirm if the sexual transmission also occurs with *M. anisopliae*, in the present study virgin males of *A. aegypti *were contaminated with conidia of two strains of *M. anisopliae*: one of low (Ma-CBG-1) and another of high virulence (Ma-CBG-2). These males were then confined with females to evaluate survival, infection rates (with insemination or not) and fecundity.

## Results

**Figure 1:**
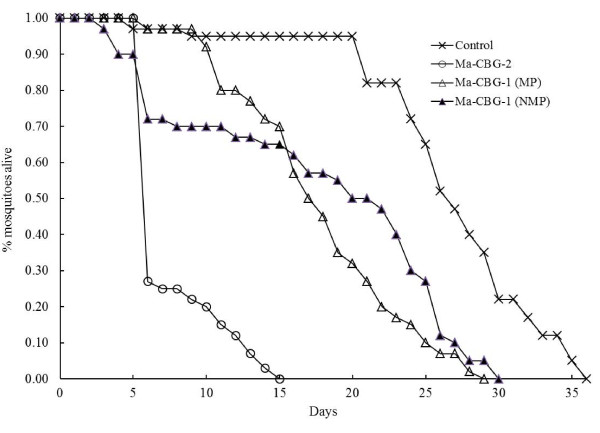
**Mean cumulative proportional survival (± Standard Error) calculated by the Kaplan-Meier model for forty females of *A. aegypti *confined with a virgin male previously exposed to 6 × 10^8 ^conidia ml^-1 ^of two isolates of *M. anisopliae *plus control (healthy male)**. Mortality by fungus was demonstrated by sporulation in cadavers (see text). Four experimental groups: the Ma-CBG-1 (NMP), Ma-CBG-1 (MP), and Ma-CBG-2 (MP) strains, and control (females with a clean male). MP = Mosquito-passaged, NMP = no mosquito-passaged.

Virulence evaluated in bioassay 1 by indirect exposure of females to filter papers impregnated with 6 × 10^8 ^conidia ml^-1 ^remained low for the NMP and MP lv strain; the LT_50 _were 18.85 (± 1.65) and 9.15 (± 0.93) days respectively. In contrast the MP hv strain showed a LT_50 _of 3.20 (± 0.70) days, and the one in the control was 25.62 (± 0.97) days (χ^2^= 28.45, df = 3, p < 0.0001). However, the susceptibility to *M. anisopliae *strains decreased even more in *A. aegypti *females confined with fungus-contaminated males. Figure 1 shows the survival curves for females confined with a male previously contaminated with both strains and the control. From these analyses the resulting LT_50 _were 21.65 (± 1.46), 17.90 (± 0.88), 7.57 (± 0.45) and 24.82 (± 0.92) days for the NMP and MP lv, MP hv, and control, respectively (χ^2^= 27.24, df = 3, p < 0.001). When comparing the mortality results from bioassay 1 and bioassay 2, virulence for the lv strain did not vary but virulence for the hv isolate decreased around 50% compared with the one obtained by indirect exposure in bioassay 1. Fungal infection also killed the males, with the hv contaminated males dying between three and six days after of fungus-exposure, while those inoculated with the lv lived up to 15 - 19 days.

Results of bioassay 2 also confirmed, by incidence of insemination, that fungal transmission from contaminated males to healthy females occurred during mating behavior. In the forty females placed together with the male contaminated with the NMP lv, there were 15 cadavers that sporulated (eight with eggs and seven with no eggs) while in cadavers with no sporulation, seven insects had laid eggs and 18 did not lay eggs before dying. Now, in those females maintained with a male contaminated with the MP lv, only nine sporulated (five with eggs and four with no eggs) and 31 not (78%), of which five laid eggs and 26 did not lay eggs. In contrast, in females in captivity with a male contaminated with the MP hv strain, 34 became sporulated (three had laid eggs and 31 laid no eggs before dying) and from the six not sporulated, none laid eggs. In the control the oviposition rate was 43% with 17 of the 40 females laying eggs. The females that managed to lay eggs but whose corpses sporulated were the ones killed by the pathogen acquired by mating with insemination. While those sporulated with no oviposition included the proportion of females killed by the fungus but transmitted through copulation attempts or other physical contacts carried out by the contaminated male.

**Figure 2:**
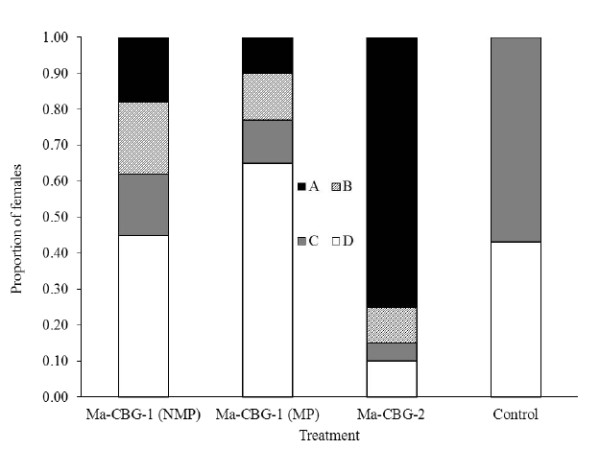
**Proportion (n = 40) of four categories of females of *A. aegypti *confined with a fungus-contaminated male, for two strains of *M. anisopliae *plus control (clean male)**. Symbols: A = Sporulated-not inseminated, B = Sporulated-inseminated, C = Not sporulated-not inseminated, D = Not sporulated-inseminated. NMP = Non-mosquito passaged, MP = mosquito passaged.

Figure 2 shows that the three NMP, MP lv and MP hv strains caused an infection rate (sporulated cadavers) of 37, 22 and 85% (15, 9 and 34/40) in females exposed to a fungus-contaminated male. According to the data, 20, 12 and 10% of mosquitoes killed by the fungi in the three treatments actually were infected through copulations with successful inseminations (true matings), while the insemination rate in the control was 46%. Overall, the analysis of the 4 × 4 contingency table constructed by treatments (columns) against groups of females was highly significant to accept the alternative hypothesis that there was a relationship between treatment and group of females (χ^2 ^= 104.78, df = 9, p < 0.0001). Therefore the proportion of inseminated and not inseminated females in each one of both groups of sporulated and not sporulated insects, varied among the four treatments.

Finally, in the three fungal treatments the pathogen transmitted by mating behavior exerted a negative impact on fecundity. The hv strain caused oogenesis to almost disappear in females (3) with a mean of 0.52 (± 0.32) eggs, which was 99% lower than the 35.35 (± 8.30) registered in control females (F = 51.88, df = 3, p < 0.001). In contrast, the lv strain, was poorly transmitted by mating behavior because all females exposed to a fungus-contaminated male (or to a clean one) died after forty days of confinement; the infection and insemination rates were 37 and 20, and 22 and 12%, for the NMP and MP isolate, respectively (χ^2 ^= 29.29, df = 3, p < 0.0001). Nonetheless, this fungal strain also reduced the fecundity to a mean of 13.65 (± 4.70) eggs, which was 40% lower than the control.

## Discussion

Both lv and hv strains of *M. anisopliae *examined here were transmitted from fungus-contaminated males to female dengue vectors. The transmission was very low following confirmed mating (10%) for the hv isolate but the sporulation rate suggests the fungus infected and killed 75% in 15 days with no insemination (Figure 2). It is unclear exactly how this high infection came about; whether it was the expression of a high rate of approaches or "strikes" that the fungus-contaminated male did with females attempting to mate with them or whether conidia were picked up by females from surfaces of the plastic container where they were confined. During the 48 hour exposure period both sexes were walking inside the container and this could have facilitated the dislodging and spreading of conidia from the male which may have led to passive transmission with the same chance of infection for all females. However, in the lv strain the rate of sporulated-not inseminated females was 17 and 12% for the NMP and MP fungus respectively, which means that the hv strain encouraged at least a four-fold increment in transmission acquired through mating attempts or simply by spores picked up from surface; in other words with the same number of conidia the lv isolate was able to initiate one infection while the hv strain was capable of four successful infections. This may also be due to the different virulence of the two strains used. The lv strain may have been less able to initiate a successful infection leading to sporulation, whereas the same level of exposure to the hv strain would be expected to lead to fungal infection, death and sporulation.

We did not register incidence of mating attempts and their associated infection rates. This component must be examined to distinguish infections caused by real copulations with insemination from infections induced by mating attempts with no insemination. Maybe more conidia are transferred in an attempt to copulate and inseminate than in the insemination itself. Insemination was more associated to no infection because the not sporulated -inseminated females were abundant (43 and 68%) in those confined with the male contaminated with the NMP and MP lv strain, while this rate was only 10% for females exposed to the hv -contaminated male.

In bioassay 1 the LT_50 _values were lower in relation to the ones of bioassay 2. Indirect exposure for 48 hours allows the females to be in contact with a major amount of conidia from the filter. In bioassay 2 the females died in longer times as a result probably from the small amounts of conidia attached to treated males. These small numbers of conidia had a lesser chance to develop infections in those females approached by males. In addition, females in bioassay 2 were blood fed and could tolerate more the invasive process of fungi as was reported previously [13,14]. Also it is important to point out that the infection rate caused by the hv strain by true mating (10%) is close to 16% (five inseminations in 30 females copulated), the insemination rate reported for virgin females confined with healthy-virgin males of *A. aegypti *in early studies [10,11]; however these old reports were registered during the first 30 minutes of confinement of both sexes in cage. In our study the fungus-contaminated male was confined for two days with females. This also suggests that the hv was so virulent that it debilitated the male to the extent that he inseminated only the 10% of the available females. Conversely, it seems that the low virulence of the lv induced in the fungus-contaminated male a higher aggressiveness for inseminating females to such an extent that the sexual activity expressed by insemination rates was somewhat higher (66%) in females confined with the fungus-contaminated male as the ones confined with the healthy male in the control (43%) (χ^2 ^= 85.34, df = 6, p < 0.0001). This may be because the immune system is activated in the lv-contaminated males and this physiological change may affect mating behaviour.

Information about autodissemination of entomopathogenic fungi by mating in hematophagous insects is little documented in the literature. The first study was carried out on the tsetse fly *Glossina morsitans morsitans *Westwood infected with *M. anisopliae *and *Beauveria bassiana *in Africa [15]; recording a 90-100% mortality in both sexes. The second report of *M. anisopliae *was for the malaria vector *Anopheles gambiae *s.l. in Africa [16], the autodissemination was of around 30% from females to males. The third report comprises results of another study [12] we conducted almost simultaneously to the present one, but assessing the transmission of two strains of *B. bassiana *by mating behavior in *A. aegypti *at the same dose of 6 × 10^8 ^conidia ml ^-1^; in that study we observed that both strains of *B. bassiana *killed 50% of the females confined with a fungus-contaminated male also in around eight days but with a transmission rate by true mating (insemination) ranging from 27 to 48%, higher than the 10% registered for the hv strain of *M. anisopliae*, however the total infection was 90% in fifteen days, a rate similar to the one observed in this study. This comparison suggests that *B. bassiana *is less aggressive to virgin males than *M. anisopliae *and allows them to be more successful in inseminating a higher number of females despite having a fungal infection.

Regarding the very low fecundity of females (0.52 ± 0.30 eggs per female) exposed to the hv -contaminated male, there are also only three related references documenting the effect of fungal infections on fecundity of vectors. In the first one, larvae of *A. aegypti *were infected with a dose of 2 × 10^5 ^conidia ml^-1 ^of *Aspergillus parasiticus *Speare [17]; the larval mortality rate was 97% but fecundity of surviving females decreased 56% in relation to the one in the control. The second study was conducted with the malaria vector *An. gambiae *[10] in which females were infected by direct exposure to 10^6 ^and 10^7 ^conidia ml^-1 ^of *M. anisopliae *with a reduction of 57-63% in fecundity. In contrast, in the present study the fecundity reduction was nearly 100% when using the hv isolate. This impact is similar to the study we conducted previously with two strains of *B. bassiana *transmitted also by mating behavior in *A. aegypti *in which both strains also reduced drastically the fecundity of females up to 96% [12]. Therefore, both *M. anisopliae *and *B. bassiana *can reduce strongly the fecundity of females exposed to the fungus-contaminated male. The youngness of females treated could explain in part the severe effect on fecundity. Two studies about *Entomophtora muscae *(Cohn) Fresenius infecting the onion fly *Delia antigua *(Meigen) [18], and the carrot fly *Psila rosae *F. [19] have reported that if the fungus infects females at the beginning of oogenesis, they are incapable of laying eggs because the pathogen incubation time is shorter than the time of oogenesis. The same was concluded in the other study where healthy females of *Musca domestica *L. were confined with *E. muscae *-sporulating cadavers of the same species; 94% of 2-5 day old females died at seven days after fungus exposure and laid 3,060 eggs, which was 19% of total eggs laid in control. However, when young, 1.5-day old females were infected, all died by day six and none laid eggs [20]. These studies were conducted by indirect exposure of flies to the fungus, however in a subsequent study the transmission by mating behavior of *E. muscae *in *M. domestica *was examined [21]; twenty 48-72 hour old, fungus-contaminated males were held together with the same number of healthy females for 24 hours for mating and then mortality and fecundity were registered daily; in the three days after confinement the fecundity of treated females was 20, 55 and 58% in comparison with the 373 eggs of healthy flies in the control (confined with clean males). They also concluded that the effect of *E. muscae *on fecundity of *M. domestica *confined with fungus-contaminated males can be not only the effect of the pathogen on females but also the damage of the fungus to the testes, when they stated "*E. muscae *-infected males are generally lethargic, lack libido and would be not competitive in nature". Therefore, it seems to be that both *M. anisopliae *and *B. bassiana *transmitted by mating exert a more profound effect on fecundity of *A. aegypti *than that of *E. muscae *on *M. domestica*.

Lastly, the MP process enhanced virulence for the lv strain where this trait was two times higher after the MP but only by indirect exposure (Figure 1). In intersexual transmission the tendency did not occur perhaps by the variable amount of conidia a male impregnated during the confinement in the exposure chambers, as was explained above. In most aggressive strains of *M. anisopliae*, host death usually occurs by the production of secondary metabolites that kill the insect after 4-16 days [22]. Production of these toxins gradually decrease as the fungus is passed through artificial media until original virulence disappears. Sometimes, but not always, the passage through insect hosts restores the virulence of these attenuated strains [23]. It has been registered that virulence of two highly pathogenic strains of *M. anisopliae *to *Culex pipiens *and *A. aegypti *larvae were significantly enhanced by one passage through a *C. pipiens *larval siphon with a relative increment from 1.63 to 2.45 times [24].

## Conclusions

*M. anisopliae *can be successfully transmitted from males to females during mating attempts. The process of passing the fungus through *A. aegypti *increased the virulence, and an entomopathogenic fungal infection was able to reduce drastically the fecundity of dengue vector mosquitoes. The hv isolate of *M. anisopliae *transmitted by males killed 85% of females in sexual encounters or through other close physical contacts after 48 hours of exposure.

This impact could be effective at reducing dengue transmission by decreasing the numbers and longevity of female mosquitoes after releasing fungus-contaminated males within dwellings. Virgin males can be contaminated with fungi by confinement in exposure chambers similar to the ones used in this study. Further studies need to be conducted to evaluate *M. anisopliae *against field populations of *A. aegypti *in order to use this pathogen as biocontrol agent of the dengue vector.

## Materials and methods

### Mosquito Colony and Fungal Handling

**Figure 3:**
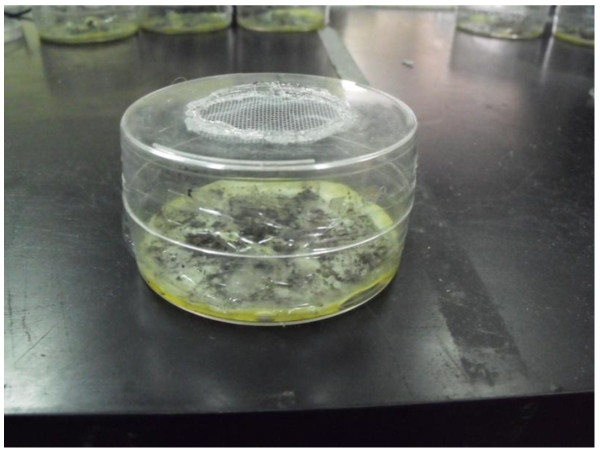
**Exposure chamber to confine *A. aegypti *adults and contaminate them with a filter at the bottom impregnated with 6 × 10^8 ^conidia ml^-1 ^of *M. anisopliae***.

Experimental insects were recently emerged males and females taken from a colony of *A. aegypti *established in 2006 with larvae from Monterrey, NL, Mexico. Mosquito rearing was carried out following the procedure of our previous report [12]. Both Ma-CBG-1 and Ma-CBG-2 strains of *M. anisopliae *were isolated from soil collected at rural habitats around the cities of Saltillo and Texcoco; then they were cultured on potato-dextrose-agar (PDA) and incubated at 25°C for 20 days to allow sporulation. For conidia harvesting a mix of 0.5% Tween 20 in 0.85% saline solution was set up: 0.5% Tween 20 was prepared by adding 5 ml of Tween in 995 ml of distilled water, while the saline solution by diluting 8.5 g of NaCl in 1 liter of distilled water, then 5 ml of the former was added to 995 ml of saline solution. Next, seven ml of the mix was added onto each plate and then the conidia layer was gently scratched from the agar. This conidia suspension was poured onto a sterile Whatman filter paper that was placed on the bottom half of a Petri plate and then dried at laboratory temperature (24°C) for 24 hours. After drying, a second half-plate was placed over the first one to create an exposure chamber (9 cm diameter, 2 cm height, 127.23 cm^3^) with a hole of 2 cm diameter cut in the top and covered with a net through which insects were introduced and removed (Figure 3).

To obtain a wide variation in virulence, the Ma-CBG-1 strain was passed five times through PDA before being passed through a mosquito host, while the Ma-CBG-2 was passed just once through PDA and then through a mosquito. To obtain these mosquito passaged (MP) versions of the fungal strains, twenty *A. aegypti *females were confined in an exposure chamber for each strain and dead mosquitoes were transferred to humid chambers for fungus incubation. The pathogen was re-isolated from sporulating cadavers, inoculated again onto PDA in Petri dishes and incubated at 25°C for 20 days. Afterwards, conidia from plates not contaminated with bacteria or other fungi were used to prepare a concentration of 6 × 10^8 ^conidia per ml of distilled water per isolate as determined with a Fisher hemocytometer. In this way we prepared exposure chambers with the least virulent (lv) Ma-CBG-1 strain for both not mosquito-passed (NMP) and MP versions, and with the highly virulent (hv) Ma-CBG-2 strain but only MP. Seven ml of the same mix Tween 20 - saline solution but with no conidia was poured onto a sterile Whatman filter paper as the control.

### Infection of Mosquitoes

Since we wanted to compare results with our previous study which used *Beauveria bassiana *[12] we used the same bioassay procedure for the two bioassays that were performed. Four treatments per bioassay were set up: the NMP and MP lv strain, the MP hv one plus a control, and two replicates of twenty females each were carried out per treatment. Bioassay 1 was conducted to estimate virulence by indirect exposure of females to both strains, and to evaluate if the mosquito-passaging increased the virulence of the lv strain. The effect of the pathogens on survival was examined in forty 4-6 day old non-blood fed females exposed to 6 × 10^8 ^conidia ml^-1 ^for 48 hours. Mosquitoes were then transferred to a 1-liter plastic pot with a cotton mesh-netting top, and mortality registered on a daily basis. Virulence was estimated as the median lethal time (LT_50_) for each treatment. Bioassay 2 was done to evaluate the effect of infection acquired from fungus -contaminated males on female survival, infection rate (inseminated and not) and fecundity. Therefore, twenty 4-6 day old, virgin male *A. aegypti *were placed into an exposure chamber for 48 hours with a filter paper impregnated with 6 × 10^8 ^conidia ml^-1 ^per treatment. Then eight males selected randomly were transferred individually to a 1-liter plastic pot with a cotton mesh-netting top and confined with twenty 4-6 day old non-blood fed females; a set of four males was a replicate, and two replicates were carried out. Clean (non-fungal contaminated) males were used in the control.

All females of each replicate were blood fed on the forearm of the same volunteer (AMGM) in the first six hours of confinement. After blood feeding insects were fed on a 5% sucrose solution offered on cotton pads and placed on the netting surface of each pot; then after around 48 hours engorged females were transferred individually to beakers half filled with distilled water and lined with filter paper for oviposition. In both bioassays, dead insects were removed daily and rinsed in 1% sodium hypochlorite for twenty seconds and then washed twice in distilled water for twenty seconds. In bioassay 2 the last two abdominal segments of all females were carefully removed immediately after death and after sterilization to check for the presence of sperm in the spermatheca which would indicate if the insemination had occurred. Ovaries were also cautiously pulled out to count retained eggs; then incomplete corpses (without the last two abdominal segments and ovaries) were placed in sterile Petri dishes lined with damp filter paper and maintained at 25°C to stimulate sporulation. Fecundity was obtained as the sum of laid and retained eggs from the first gonotrophic cycle.

### Statistical Analyses

The median lethal time (LT_50_) was obtained from the survival analysis computed with the Kaplan-Meier model for the forty females per treatment in both bioassays. The infection-insemination rates were analyzed by a 4 × 4 treatment -group contingency table, which was built by counting the number of females from the forty in each one of the four treatments across four groups: sporulated-inseminated females, sporulated-not inseminated ones, not sporulated-inseminated, and not sporulated-not inseminated mosquitoes. In the control there were only inseminated and not inseminated females. So, the four treatments in the columns were the populations, and groups of females per treatment were the variables [25]. Afterwards, a test of independency as null hypothesis was performed based on the χ^2 ^statistic with proc freq in SAS. The mean fecundity of mosquitoes exposed to the different treatments was analyzed by ANOVA for unbalanced experiments and Ryan test as *post hoc *multiple mean comparison was also computed with proc glm in SAS [26].

## Competing interests

The authors declare that they have no competing interests.

## Authors' contributions

FRV conceived the study, the experimental design and conducted the statistical analyses. JAGH helped to carry out the experiments, AMGM participated in the maintenance of the

insect colonies. PTG participated in the design of bioassays, AFVH participated in the data analyses and in writing the final manuscript, MARP participated in the design, supervised the experiments and wrote the first manuscript. All authors read and approved the final manuscript.
